# Histological and Immunohistochemical Methods in Normal and Osteoarthritic Knee Cartilage of Rat and Rabbit Models: A Literature Review

**DOI:** 10.3390/ijms262110300

**Published:** 2025-10-23

**Authors:** Ana Sabucedo-Suárez, María Permuy, Fernando Muñoz, Mónica López-Peña

**Affiliations:** 1 Anatomy, Animal Production and Veterinary Clinical Sciences Department, Veterinary Faculty, Universidade de Santiago de Compostela, 27002 Lugo, Spain; anamaria.sabucedo@rai.usc.es (A.S.-S.); maria.permuy@usc.es (M.P.); fernandom.munoz@usc.es (F.M.); 2 Laboratory of Biomaterials, Ibonelab S.L., 27004 Lugo, Spain

**Keywords:** articular cartilage, osteoarthritis, immunohistochemistry, histology, rabbit, rat

## Abstract

The tissue covering the bones in synovial joints is called articular cartilage. Chondrocytes produce and maintain the extracellular matrix and, based on their shape and the orientation of the collagen fibers, articular cartilage is separated into four histological zones: superficial, middle, deep, and calcified zones. Osteoarthritis is a degenerative joint disorder in which mechanical, biochemical, and inflammatory factors contribute to the disruption of the balance between extracellular matrix synthesis and degradation. This article aims to review the literature published to date by identifying the techniques most used in immunohistochemistry and histology for the detection and grading of knee osteoarthritis in rabbit/rat models. A systematic review was carried out using databases to find publications that assessed osteoarthritis in rabbit/rat knee models using histological and immunohistochemical methods. Out of 766 initial articles, 56 met the criteria. Hematoxylin–Eosin and Safranin O demonstrated clear distinctions between healthy and osteoarthritis cartilage. Immunohistochemical findings showed decreased expression of type II collagen and increased expression of matrix metalloproteinases and caspase-3 in osteoarthritis cartilage. Although both have limitations, histology stains are useful for evaluating cartilage structure and osteoarthritis progression. On the other hand, immunohistochemistry techniques support established osteoarthritis processes, including apoptosis, metalloproteinases activity, and collagen degradation. Future research should explore additional pathways to improve osteoarthritis understanding.

## 1. Introduction

Articular cartilage (AC) is a specialized organ that encases the bony components that make up the synovial joint. Its main characteristic is resilience, allowing it to withstand years of cyclic stress by supporting loads and absorbing impact. These properties are related to the composition and structure of its extracellular matrix (ECM) [1].

The ECM comprises several types of collagen, of which type II collagen (COL-II) accounts for up to 90–95% [2]. These collagen fibers form a network that envelops proteoglycans, water, and glycoproteins [3].

AC has a single cell line, the chondrocytes, which is responsible for the synthesis and maintenance of ECM [4]. The chondrocytes’ morphology, together with the orientation of the collagen fibers, allows us to divide AC into four histological zones, which are ordered from the most distal to the bone to the most proximal: the superficial (tangential), middle (transitional), deep (radial), and calcified zones [5].

**Figure 1:**
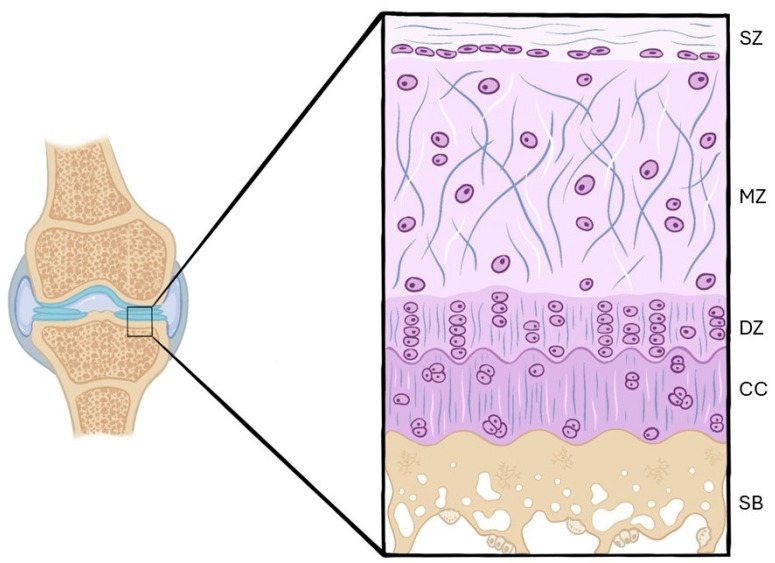
Layers of the normal articular cartilage. SZ—superficial zone; MZ—middle zone; DZ—deep zone; CC—calcified cartilage; SB—subchondral bone.

The superficial zone is the thinnest layer. The chondrocytes have an elongated shape, and the collagen fibers are arranged parallel to the joint. The middle zone has less cell density, and the chondrocytes are oval and placed in small groups. In this layer, the collagen fibers change their arrangement to lie obliquely to the joint. In the deep zone, the chondrocytes organize columns, and the collagen fibers are perpendicular to the joint. Finally, the calcified zone has a very low cell population, and the chondrocytes hypertrophy and synthesize fibrocartilage, which will act as cushioning between the AC and the subchondral bone [1] (Figure 1).

**Figure 2:**
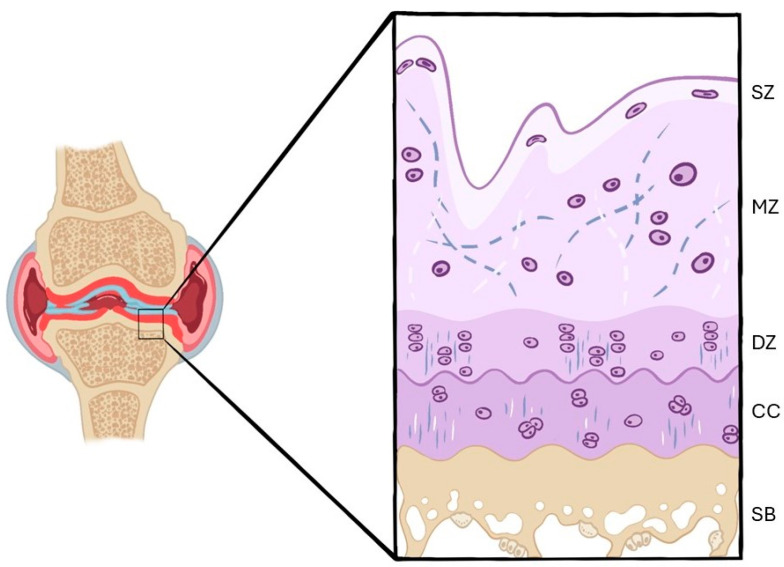
Changes in the articular cartilage in the presence of OA. SZ—superficial zone; MZ—middle zone; DZ—deep zone; CC—calcified cartilage; SB—subchondral bone. Progressive structural disorganization, loss of chondrocytes, and disruption of the collagen fiber network, together with subchondral bone thickening and sclerosis can be observed. These histological alterations illustrate the typical zonal progression of osteoarthritic degeneration.

In a non-pathological state, there is hardly any exchange and activation of chondrocytes in ECM. However, when a disorder starts, such as osteoarthritis (OA), mechanical and biochemical changes will result in an imbalance between ECM synthesis and degradation [6]. That is, chondrocytes become activated and start producing both proteins and enzymes involved in its degradation. Therefore, in the first phase, a hypertrophic reaction will appear, increasing ECM synthesis and content. This is followed by a stage of high replacement of the matrix, leading to a depletion of its components. Finally, the collagen network is damaged and lost. At this point, typical OA lesions appear [7] (Figure 2).

Initially, the enzymes responsible for this degradation include aggrecanases and matrix metalloproteinases (MMPs) (for aggrecan and COL-II, respectively). In this way, the collagen network is degraded to a point where damage is irreversible [6]. Moreover, inflammatory mediators are released from the synovial tissue, mainly interleukin 1β (IL-1β) and tumor necrosis factor α (TNF-α), which further increase proteinase production [8]. In addition, IL-1β simultaneously suppresses COL-II synthesis, increases collagen type I synthesis, and reduces aggrecan synthesis. Thus, IL-1β reduces anabolic activity while increasing catabolic activity [9].

As the process progresses, ECM degradation results in the loss of survival mechanisms, calcification, and apoptosis. This apoptosis of cartilage or chondrocytes can be mediated by different kinds of caspases, of which caspase-3 plays a relevant role in mediating this phase [10].

OA is the most prevalent chronic joint disease and a leading cause of pain and disability worldwide. It is a multifactorial and complex condition influenced by a combination of biomechanical, metabolic, and biological factors. Occupational load, intense sports participation, previous joint injuries, obesity, and sex-specific physiological differences are recognized risk factors that contribute to its onset and progression. Early detection is crucial for effective management, yet clinical evaluation remains largely subjective and limited in sensitivity during the initial stages of disease [11]. These challenges underscore the importance of translational and preclinical studies that elucidate the underlying histological and molecular mechanisms of cartilage degeneration.

The study of osteoarthritis in preclinical models such as rats and rabbits has also enabled the identification of a broad variety of cellular mechanisms involved in disease progression [12]. Histological techniques not only provide quantitative and qualitative assessment of cartilage damage but also help characterize features such as chondrocyte proliferation, loss of proteoglycans, and cell clustering that are associated with degenerative and reparative phases of OA [13]. For example, in normal cartilage, Safranin O–Fast Green and Toluidine Blue staining are widely applied for evaluating matrix proteoglycans, while in OA tissue they allow visualization of proteoglycan depletion, fibrillation, and tidemark duplication. Hematoxylin–Eosin is often used for general tissue structure and cellular morphology [14]. By applying standardized scoring systems, such as Mankin and OARSI, these histological changes can be correlated with disease stage and with the predominance of different cell types throughout OA pathology [15].

Immunohistochemistry has permitted us to identify the expression of a wide variety of molecules and their distribution in normal or pathological AC [16], improving the knowledge of the disease. In healthy cartilage, immunostaining commonly targets type II collagen and aggrecan, whereas in OA cartilage the overexpression of degradative enzymes or hypertrophic markers is highlighted to illustrate pathological remodeling [17].

The use of markers in osteoarthritis allows the identification of key processes such as degradation, inflammation, and apoptosis, and provides functional and spatial maps of different types of joint cells, thereby facilitating the analysis of the multifactorial nature of OA and the specific mechanisms depending on the model and the type of intervention used [18]. Thus, correlating the expression of these markers with histological findings helps to deepen the understanding of the underlying cellular mechanisms, highlighting how these techniques contribute to advancing knowledge on the pathophysiology of osteoarthritis.

In short, OA is a multifactorial process involving multiple pathways, many of which are not yet fully understood. This review is specifically focused on rat and rabbit models, as they represent two of the most widely used preclinical models in the study of OA pathology/pathophysiology, and assessing the effectiveness of different therapeutics [19]. These species provide important insights into the histological and immunohistochemical features of OA while allowing controlled experimental manipulation. The scope of the present article is therefore limited to studies in rats and rabbits to summarize methodological approaches in these models. By doing so, we intend to highlight the preclinical methodological framework that supports translational advances in osteoarthritis research.

## 2. Materials and Methods

The articles included in this publication were manually reviewed in early 2025.

### 2.1. Search Strategy

The search of publications for this review was performed through a series of databases: PubMed, Scopus, and Web of Science (WOS) in January 2025. Different permutations of the following terms were used to identify these publications: “OA”, “animal models”, “rat”, “rabbit”, “immunohistochemistry”, and/or “knee joint”. Finally, this research included articles published from 2003 to 2024.

### 2.2. Inclusion and Exclusion Criteria

#### 2.2.1. Inclusion Criteria

Articles in English.

Studies performed on rabbits.

Studies performed on rats.

The presence of knee OA.

Immunohistochemistry evaluation.

Comparison between normal and osteoarthritic cartilage.

#### 2.2.2. Exclusion Criteria

Reviews.

Abstracts.

Articles in a language other than English.

Studies on different joints rather than knee joints.

Studies on different animal species rather than rabbits or rats.

No data of histological and immunohistochemistry evaluation present in the study.

No presence of normal cartilage evaluation.

### 2.3. Screening Method and Data Extraction

There were two phases to the screening process. Initially, each article’s title and abstract were chosen from the various database search results. Then, the duplicates were removed, and the inclusion and exclusion criteria were used for independent screening by two reviewers, A.S. and F.M.

The same two reviewers (A.S. and F.M.) examined the full text of the articles for eligibility. Relevant data were examined and extracted: animal model, the OA location, the histological and immunohistochemistry techniques, as well as the positive or negative outcomes for each study.

Finally, the inclusion and exclusion criteria helped in the selection of the final studies included.

## 3. Results

### 3.1. Study Selection

**Figure 3:**
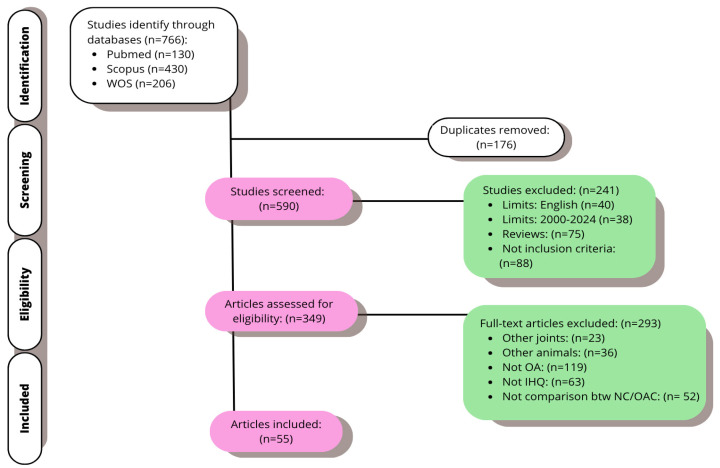
Flow chart of the final selection.

From the databases, the initial research yielded 766 articles. The duplicates were removed; the remaining 590 were screened. The exclusion/inclusion criteria indicated in the third section of this review were used, and the title and abstract were examined. At this point, 293 items in all were disqualified. Ultimately, 55 publications from the full-text analysis of 349 investigations were chosen for this study (Figure 3).

### 3.2. Main Outcomes

The results of this review are represented in Table 1 for rabbits and Table 2 for rats, which analyze the 55 selected articles, with the histology and immunohistochemistry techniques used in each one.

#### 3.2.1. Histological Findings

**Figure 4:**
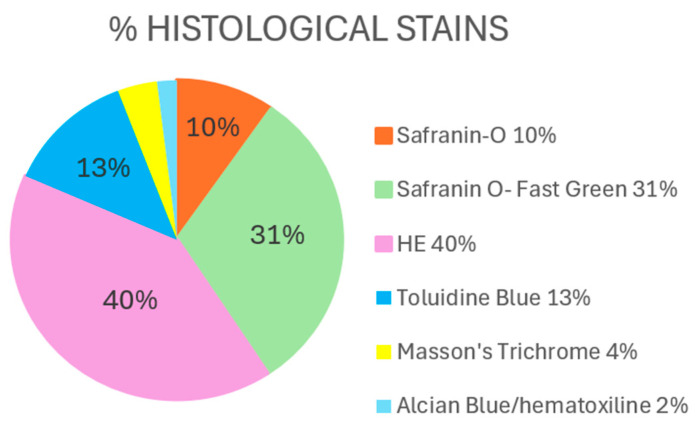
Percentage of most commonly histological stains used by the authors. Hematoxylin–Eosin (HE) and Safranin O–Fast Green, representing 40% and 31%, respectively, were the most frequently applied, reflecting their widespread utility for general morphological and glycosaminoglycan assessment, respectively. Toluidine Blue (13%) was also extensively used to evaluate proteoglycan content.

The techniques most commonly used by all the authors were Hematoxylin–Eosin (HE), followed by Safranin O (SO) with or without Fast Green (SO-FG) and Toluidine Blue (TB) (Figure 4).

##### Hematoxylin–Eosin

- Control groups (healthy knees)

Both experimental models describe the structure of articular cartilage in healthy knees with similar characteristics. In rabbits, virtually all studies agree on a smooth surface, chondrocytes arranged in an orderly manner and of similar size, a uniformly stained extracellular matrix (ECM), and an intact tide line [25,31,34,71,73,74].

In the same way, smooth articular surfaces and normal tissue architecture have been reported in rats; for example, Aktas et al. (2011) [38], Wen et al. (2013) [41], and Gökdemir et al. (2024) [64] report cartilage with intact surfaces and regular distribution of chondrocytes, while Assis et al. (2016) [45], Moon et al. (2012) [39], and Giunta et al. (2024) [72] describe a parallel and columnar arrangement of chondrocytes. Finally, Wen et al. (2013) [41] describe the flattened appearance of these cells.

- OA groups (OA knees)

Despite some particularities, associated with the species or the experimental method, the histological changes associated with osteoarthritis have been consistent in both models. In rabbits, OA groups are characterized by irregular surfaces, the presence of fissures, and disorganization in the arrangement of chondrocytes [28,29,32,35]. In addition, some authors reported the presence of fibrillation [23,24] and the formation of chondrocyte clusters [26], as well as a thinning of the chondrocyte layer and reduction in cell number compared with controls [25,33,34,74]. The tide line is described in some studies as destroyed [37] and in others as present but irregular [31].

In rats, OA models show similar alterations. Aktas et al. (2011) [38] identified discontinuities in the matrix, with vertical and branching fissures extending from the superficial to the medial zone. Complementarily, Assis et al. (2016) [45] and Moon et al. (2012) [39] reported tissue degradation and intense fibrillation.

Bei et al. (2019) [53] and Di Rosa et al. (2014) [42] described the presence of horizontal fissures, chondrocyte swelling, erosions, and fibrous tissue proliferation, resulting in cartilage thinning and damaged tide line integrity. Furthermore, Gökdemir et al. (2024) [64], Han et al. (2024) [68], and Wang et al. (2020) [56] complemented these findings by reporting irregular surfaces, a reduction in tissue thickness, and a decrease in the number of chondrocytes, while Wei et al. (2017) [48], Wen et al. (2016) [46], and Yan et al. (2018, 2019) [51,54], together with Sun et al. (2023) [67], emphasized marked cell loss and general degeneration of cartilage.

Some studies have evidenced modifications, such as duplication or disappearance of the tide line and the presence of clusters or ghost cells in areas with progressive damage [52,69]. Moreover, Yang et al. (2020) [57] reported significant cartilage erosion accompanied by subchondral bone remodeling, and Zhu et al. (2021) [60], together with Chen et al. (2021) [61], documented regional loss of matrix and chondrocytes, with osteophyte formation in deteriorated areas. Finally, additional investigations [36,65,66,71] complement the picture by pointing out the presence of edema, superficial abrasion, pannus formation, and fissures reaching the subchondral bone.

##### Safranin O/Safranin O–Fast Green

- Control groups (healthy knees)

Studies in rabbits almost unanimously describe that, in control groups, SO-FG reveals uniformly red-stained cartilage and green-stained subchondral bone, facilitating the clear identification of tissue morphology and distribution [6,25,30,34,73]. Similarly, in rats, it has been evidenced that controls exhibit a smooth articular surface with intense and homogeneous matrix staining, showing abundant proteoglycans and a correct distribution of chondrocytes [39,40,64,66].

These results reflect that, under normal conditions, both rabbits and rats present a characteristic histological pattern with a clear differentiation between cartilage matrix and subchondral bone.

- OA groups (OA knees)

In rabbits, most authors have observed a decrease in intensity and uniformity of staining with Safranin O or Safranin O–Fast Green. These findings suggest a loss of proteoglycan content in the matrix [21,24,25,26,27,28,34]. Other studies, however, used this staining to describe cartilage morphology, obtaining similar results to those described by HE [6,20,22,30,50]. Nevertheless, Shirai et al. (2011) [24] found no significant differences between the groups.

In rats, several studies have documented findings consistent with cartilage degradation in OA models. Similar to what happened in rabbits, there are some studies that focus primarily on describing the loss of proteoglycan content and the reduction in staining intensity, while others point to morphological and structural alterations.

Therefore, Chen et al. (2018) [50] and Murata et al. (2017) [47] reported significantly lower relative GAG density in the OA group compared with the sham/intact group. And similarly, Lee et al. (2013) [40] and Moon et al. (2012) [39] observed that the reduction in proteoglycan content was accompanied by a decrease in cartilage thickness.

Meanwhile, Barreto et al. (2022) [63] described, in the OA group, poor impregnation, swollen chondrocytes, reduction in their number, and irregularities in distribution, with an evident loss of the matrix component. Bei et al. (2019) [53] also noted surface irregularities, with fissures, chondrocyte swelling, and denudation, in contrast with controls showing only a slight decrease in staining. Gökdemir et al. (2024) [64] and Luo et al. (2021) [59] described that, while in controls, cartilage structure and thickness were normal, cartilage thinning, loss of uniformity in staining, and alterations in chondrocyte arrangement were evident in the OA group.

Additional studies [46,51,56,58,62,67] have corroborated these findings, showing that a marked decrease in proteoglycans, collagen disorganization, and overall loss of normal matrix patterning is observed in OA models. Chen et al. (2021) [52] also highlighted that the number of chondrocytes is drastically reduced in OA groups, accompanied by an increase in apoptotic chondrocytes. Finally, Song et al. (2022) [65], Chen et al. (2022) [36], and Lee et al. (2024) [70] reported the presence of edema, erosion, and deep lesions evidencing severe cartilage damage in OA models, concluding that, in some cases, almost complete absence of staining can be found in damaged areas [66,70,71].

##### Toluidine Blue

- Control groups (healthy knees)

In rabbit studies, although the description focuses on the loss of intensity in the OA groups, it is understood that, in healthy conditions, the TB staining shows marked metachromasia, indicating the normal presence of proteoglycans in the cartilage matrix [33,34]. Similarly, in rats, controls exhibit a smooth articular surface with a homogeneous distribution of chondrocytes and a proteoglycan-rich extracellular matrix [43,49,52].

- OA groups (OA knees)

Rabbit studies have reported a decrease in staining intensity in the OA groups, suggesting a loss of proteoglycan in articular cartilage [33,34].

In rats, Lijima et al. (2015) [43] described an articular surface with fibrillation and a disrupted medial region of the cartilage, where significant proteoglycan wasting was evident. Similarly, Lijima et al. (2016) [44] confirmed proteoglycan loss and evidence of subchondral bone damage. Ping et al. (2020) [55] and Wei et al. (2017) [48] reported erosions in the medial cartilage layer and a marked reduction in the number of chondrocytes in the OA groups, which was reflected in decreased staining intensity.

Moreover, Gökdemir et al. (2024) [39] found that, in the OA group, degeneration extended to the lower layers, with changes in cell distribution and thinning of the cartilage. Isaka et al. (2017) [49] documented irregularities on the surface of the medial femoral condyle and tibial plateau, along with a marked reduction in matrix staining. Finally, Jacer et al. (2018) [67] evidenced the loss of metachromasia in the ECM of the OA group, replaced by uniform blue staining, which was interpreted as a histological sign of cartilage destruction and formation of a fibrocartilage layer.

##### Others

Finally, concerning the remaining techniques commonly used, in Masson’s trichrome (MT), damage was observed to the collagen of the articular cartilage [32,35].

In sections stained with Alcian Blue/Hematoxylin, remarkable cartilage degeneration was observed in OA models. Yan et al. (2022) [62] reported a loss of chondrocytes, glycosaminoglycans, and disorganization of the collagen matrix. These findings were confirmed by the studies of Yan et al. (2018, 2019) [51,54], where chondrocyte apoptosis, collagen depletion, and altered tissue organization were also described. Likewise, Haleem et al. (2024) [69] evidenced that, under OA conditions, chondrocytes presented a faint staining in contrast with the intense staining observed in the control group.

It should be noted that the use of Sirius Red by Zhou et al. (2015) [27] allowed them to analyze the alignment of collagen fibers, as well as their diameter and birefringence (i.e., light intensity) with polarized light. In this case, a gradual increase in the disruption of birefringence was described with the progression of OA.

#### 3.2.2. Immunohistochemical Findings

**Figure 5:**
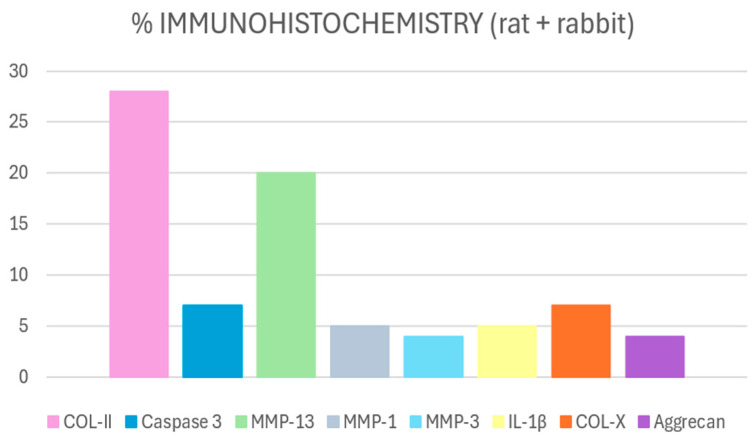
Percentage of most used immunohistochemical markers in rat and rabbit models of osteoarthritis. Collagen type II (COL-II) was the most frequently assessed, reaching 28%, reflecting extracellular matrix integrity, followed by matrix metalloproteinase-13 (MMP-13) (20%), a key enzyme in cartilage degradation. Caspase-3 was used in 7% of the studies to evaluate chondrocyte apoptosis.

Of all markers used in the articles included in this review, the most used are collagen type 2 (COL-II) and caspase-3, closely followed by metalloproteins (MMP), mainly 13, but also 1 and 3, and collagen type X (COL-X) (Figure 5).

##### COL-II

Studies agree that COL-II expression is high in healthy tissues and significantly reduced in OA ones [27,30,31,34]. Cheng et al. (2018) [50] reported that COL-II density was significantly decreased in the OA group compared with the sham group. Similarly, a lot of authors described a marked decrease in COL-II in impaired cartilage versus control groups [36,44,49,52,53,57,60,61,63,65,66,69,70,71].

##### MMP-13

In contrast, MMP-13 expression is markedly increased in OA cartilage [21,25,32,34]. Assis et al. (2016) [45] detected higher MMP-13 immunoexpression in OA samples than in controls. Similarly, Lee et al. (2013) [40] and Moon et al. (2012) [39] observed an increase in the number of MMP-13-positive chondrocytes after the induction of OA. In addition, Ping et al. (2020) [55], in conjunction with other authors [36,51,54,57,60,61,66], reported little or no immunopositivity in normal cartilage versus strong expression in OA models. For Shirai et al. (2011) [24], there was no difference between both groups.

##### Caspase-3

Activation of caspase-3 is low in healthy cartilage and increases in OA [6,74]. Similarly, Assis et al. (2016) [45] and Murata et al. (2017) [47] pointed out that the positive area for caspase-3 was significantly increased in the OA group compared with intact tissues.

##### COL-X

COL-X (a marker of hypertrophy and abnormal cartilage maturation) is expressed at low levels in healthy tissue and increases in OA. Cheng et al. (2018) [50] evidenced that COL-X was predominantly localized in chondrocytes in the OA group, and Ping et al. (2020) [55], Yan et al. (2018) [51], Zhu et al. (2021) [60], and Chen et al. (2021) [61] reported a significant increase in COL-X in OA models versus controls.

##### Other Markers

Other studies have evaluated various mediators involved in cartilage degradation and inflammatory response. For example, Aktas et al. (2011) [38] and Bei et al. (2019) [53] reported an increase in MMP-3 in OA groups, while Assis et al. (2016) [45] and Murata et al. (2017) [47] examined inflammatory markers such as IL-1β and TNF-α, finding elevated levels in OA groups. Likewise, iNOS, nitrotyrosine, RAGE, NF-κB, BMP-2, PACAP, COL-I, MMP-8, MMP-9, ADAMTS-4/5, TGF-β1, proliferation (PCNA), and autophagy (LC3) markers have been evaluated. Taken together, these findings reflect a pattern in which, in OA models, the expression of degradative proteases and inflammatory mediators increased, while cartilage structural components decreased, evidencing the pathogenetic complexity of the disease [42,46,56,59,62,64,65,68,70,71,72].

##### Difference According to the Animal Model Used

**Figure 6:**
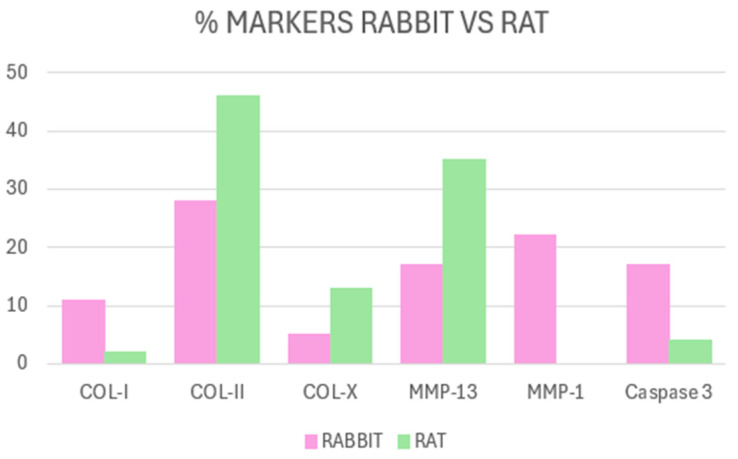
Difference between markers used in rabbit studies vs. rat studies included in this article. In both species, collagen type II (COL-II) was the most frequently assessed. In rats, MMP-13 was the second most analyzed marker, whereas, in rabbits, MMP-1 predominated. Collagen type I (COL-I) and caspase-3 were used more extensively in rabbits than in rats.

Comparing the results of the rabbit and rat articles, we observe that COL-II is indisputably the most used in both models [27,30,31,34,36,40,43,44,49,50,51,52,53,54,55,56,57,59,60,61,62,63,65,66,68,69,71,74]. However, COL-I is used more in rabbits than in rats, being 11% of rabbit studies [27,34] versus 2% in rats [38]. Similarities occur with the use of caspase-3, which represents 17% of the articles included in this review in the rabbit model [6,33,74] versus 4% in rats [45,47]. Finally, the use of MMP-1 in rabbits represents 22% of the articles [20,21,32,35], but, in rats, its use completely disappears (Figure 6). These differences may reflect species-specific biological characteristics or methodological variations related to the induction models employed, as further discussed in the following section.

## 4. Discussion

### 4.1. Histology

For the detection and histological evaluation of osteoarthritis in both rat and rabbit models, the results of this review indicate that three stains are mostly used: HE, SO-FG, and TB. Together, they allow the analysis of both cartilage integrity and changes in its extracellular matrix.

HE staining allows the assessment of chondrocyte morphology and distribution changes, indicative of cellular proliferation or death occurring during disease advancement [25,31,33,34,37,74].

As for Safranin O, with or without Fast Green, it is used mainly as an indicator of proteoglycan and glycosaminoglycan (GAG) loss in osteoarthritic cartilage [21,23,25,26,27,28,34], reflecting enzymatic digestion and matrix breakdown processes [75]. Therefore, it is crucial always to compare a control group with a pathologic or osteoarthritic group, showing the loss of intensity in the staining.

Furthermore, binding to GAGs and PGs by these two stains only occurs when the amount of these components is relatively high, which limits their sensitivity in cases of severe deterioration, i.e., advanced OA processes [76]. Because of this, Gerwin et al. (2010) [75] advise against its use for quantifying staining intensity by histomorphometry.

Laverty et al. (2010) [77] also point out that if plastic embedded material is used to process the samples and obtain the sections for histological evaluation, these methods are not completely reliable as they may underestimate the proteoglycan content.

It is noteworthy that the articles that did not use HE for the structural description of cartilage but used Safranin O or Safranin O–Fast Green, obtaining similar descriptions to the OA groups [6,20,22,30].

As we have seen, each stain has its strengths but also its limitations. That is because Cook et al. (2010) [78] propose that an appropriate protocol should include three phases: HE for the evaluation of the general cartilage structure, TB or SO for PG detection, and finally, Picrosirius Red with polarized light for the collagen analysis. Although both TB and SO can reveal PG distribution, SO is generally considered more specific and sensitive for evaluating and quantifying proteoglycan depletion due to its higher specificity and contrast, whereas TB provides complementary metachromatic staining that facilitates morphological assessment.

Therefore, when we complement these advanced techniques with each other, the comprehensive histological evaluation becomes a powerful tool to correlate structural changes with cellular behaviors such as apoptosis and matrix remodeling, which are crucial in OA pathophysiology [78]. These capabilities underscore why the selected staining protocols are prevalently used in rat and rabbit models to elucidate the multifactorial and dynamic nature of osteoarthritis.

To sum up, histological techniques, standardized through OARSI recommendations for rabbit OA models [77], provide detailed and semi-quantitative evaluations of joint lesions in cartilage, bone, and synovium. Their main strength lies in consistently reproducing a wide range of OA-associated structural changes, facilitating comparisons across studies and research centers, and enabling the assessment of experimental therapies in multiple joint tissues. However, these methods have limitations, including the subjectivity of scoring systems, the labor-intensive nature of sample preparation and staining, and their invasive character, which precludes dynamic follow-up in living subjects.

### 4.2. Immunohistochemistry

In general, except for Shirai et al. (2011) [24], the results obtained by the studies allow us to confirm the theory that we know so far about the pathways that play a principal role in the pathology of OA.

Collagen fibers are damaged and lost as the pathology progresses, and, immunohistochemically, collagen type II expression decreases in OA groups [27,30,31,34]. MMPs, on the other hand, are responsible for this degradation of collagen fibers [21,25,32,34]. And finally, a good indicator of the final stages of OA is caspase-3, which is one of the mediators of chondrocyte apoptosis [6,74].

Therefore, the selection of immunohistochemical markers responds to the need to analyze specific processes at the molecular level, such as those just mentioned. The most informative markers are those of cartilage metabolism, as they most accurately reflect cartilage degradation, and are also the most widely used, typically found at the end of tissue destruction pathways (such as COL-II) [79].

Beyond confirming the involvement of collagen degradation, matrix metalloproteinases, and apoptosis mediators in osteoarthritis progression, immunohistochemical techniques provide critical insights into the underlying inflammatory and catabolic pathways active within affected joint tissues [80]. The decreased expression of collagen type II reflects the loss of cartilage matrix integrity, while the heightened presence of MMPs, particularly MMP-13, indicates active enzymatic breakdown of collagen fibers driven by inflammatory cytokines such as IL-1β and TNF-α. Caspase-3 expression signals ongoing chondrocyte apoptosis contributing to cartilage degeneration. These markers collectively inform on the dynamic interactions between cellular stress, matrix remodeling, and inflammation characteristics of OA pathophysiology. Variations in marker expression observed between rat and rabbit models highlight the impact of species differences and OA induction methods on disease mechanisms, emphasizing the need for tailored interpretation within each experimental context. Nonetheless, immunohistochemistry remains indispensable for elucidating molecular events driving osteoarthritis and validating targets for therapeutic intervention [80].

Comparing both human OA cartilage and preclinical models, COL-II degradation signifies the loss of cartilage matrix integrity; however, the extent and temporal dynamics of COL-II expression may differ due to species-specific cartilage composition and turnover rates. MMP-13, a principal matrix metalloproteinase involved in collagen breakdown, consistently shows upregulated expression across humans, rabbits, and rats, indicating a shared catabolic mechanism driving cartilage degeneration [81]. Caspase-3-mediated apoptosis of chondrocytes is similarly observed in all species, yet discrepancies in the onset and intensity of caspase-3 activation reflect differences in disease progression and experimental OA induction methods [82]. Understanding these interspecies variabilities allows for a more nuanced interpretation of immunohistochemical data and better alignment of animal model results with the clinical pathophysiology of human OA.

It is also important to consider that the differences observed between rabbits and rats in the use of certain immunohistochemical markers may be related to species-specific physiological characteristics and the OA induction models commonly employed.

In rats, one of the most frequently used methods is the intraarticular injection of sodium monoiodoacetate (MIA) to induce OA. McCoy et al. (2015) [83] describe that this technique leads to abrupt and reproducible joint degeneration within a short timeframe, potentially altering the timing of peak expression for some biomarkers and complicating their immunohistochemical detection during intermediate phases.

For instance, caspase-3 shows sustained expression between 2 and 4 weeks in rabbits [84]. Also, in rats, a knee OA surgically induced model described the increase in caspase-3 expression at 2 and 4 weeks [45,47]. In contrast, Guo et al. (2022) [85] report a sharp peak in caspase-3 expression just three days after MIA induction, followed by a marked decline by day seven. These dynamics suggest that caspase-3 may have a limited utility as a marker in this model due to its transient expression.

A similar trend was observed for type I collagen. In rabbits, its expression increases until week 6 [33], followed by a gradual decrease at weeks 8 and 16 [28,34]. In rats, however, studies report a decrease as early as 2–4 weeks post-induction [59,86].

Regarding MMP-1, its expression has been well documented in rabbits, showing a progressive increase up to 8 weeks post-induction [87]. In rats, however, none of the studies included in this review utilized MMP-1 as a marker, suggesting that it is not a common target in this species, although its actual expression cannot be ruled out due to the lack of studies.

These interspecies variations highlight the importance of comparative analysis for translational research and go beyond simple methodological differences. Researchers can determine which pathways, cellular reactions, and biomarker dynamics may most accurately represent the clinical variability seen in human patients by examining how OA arises and advances in both rabbits and rats. The impact of joint structure, metabolism, and repair potential on disease development and biomarker interpretation is exemplified by species-specific differences in the timing and intensity of caspase-3, collagen I, or MMP-1 expression. Therefore, thorough histological and immunohistochemical analysis in both models enhances the clinical relevance of experimental research in osteoarthritis by optimizing preclinical study design and facilitating the identification of therapeutic targets and prognostic markers.

Taken together, these considerations highlight that immunohistochemistry provides deeper insight into the molecular mechanisms driving osteoarthritis progression. It is particularly valuable for characterizing cell populations, detecting markers of inflammation, apoptosis, and matrix degradation, and evaluating therapeutic responses. Nonetheless, this technique has notable limitations, including the reliance on antibody quality and specificity, signal variability, and potential interpretative bias, as well as higher cost and technical complexity compared with conventional histology [77].

Looking ahead, the application of advanced imaging techniques, such as polarization-resolved second harmonic generation microscopy, has revolutionized the exploration of cellular components and the extracellular matrix of articular cartilage. As demonstrated in recent studies, including those by He et al. (2014) [88] and Wu et al. (2017) [89], these methodologies enable high resolution visualization and analysis of the multilaminar architecture and collagen organization, as well as the mechanical and pathological effects on the tissue. In this way, the acquisition of functional and structural parameters of cartilage ultrastructure provides new opportunities for early diagnosis and dynamic monitoring of osteoarthritis, enhancing their future implementation in clinical trials and translational practice as highly specific non-invasive tools for pathology characterization and therapeutic follow-up.

Furthermore, advances in digital histomorphometry and artificial intelligence are reshaping histological evaluation in osteoarthritis. AI-assisted image analysis enables automated objective quantification of cartilage matrix degradation and cellular organization, improving reproducibility and minimizing observer bias [90]. Deep learning algorithms trained with large annotated datasets have shown remarkable accuracy in grading osteoarthritic lesions and identifying early microstructural changes invisible to manual scoring [91]. Integrated within digital pathology frameworks, these tools enhance diagnostic precision and accelerate translational research by enabling high throughput multiparametric tissue analysis.

### 4.3. Limitations

This review has several methodological constraints that should be considered. First, it does not include a formal meta-analysis, which limits the ability to quantitatively synthesize and compare outcomes across heterogeneous experimental designs. Consequently, the conclusions drawn rely mainly on descriptive and comparative interpretation of published evidence. Second, variability among animal models, histological grading systems, and staining protocols introduces potential bias and reduces inter-study comparability. The inherent subjectivity of histological interpretation remains another limitation despite the use of semiquantitative scoring systems, as differences in observer experience can influence outcome evaluation. We therefore recommend that the results of this review be interpreted with caution and with these limitations in mind.

## 5. Conclusions

Staining techniques such as Hematoxylin–Eosin (HE) and Safranin O (SO), along with Fast Green (SO-FG), are useful for analyzing the presence and progression of osteoarthritis (OA), although each has its limitations. HE allows observation of the general structure of cartilage, differentiating between healthy and pathological tissues, but does not provide detailed information. SO, on the other hand, requires a control sample to compare the loss of intensity and detect cartilage degradation.

In animal models such as rats and rabbits, HE has demonstrated clear differences between healthy and damaged cartilage, showing changes such as cellular disorganization, tissue thinning, and alteration of the tidemark in cases of OA. Staining with SO and TB has also been shown to be effective in evidencing proteoglycan loss and structural alterations, although they have limitations at low concentrations of PG, so it would be interesting to improve their reliability in advanced stages of the disease.

However, we believe that, due to the pros and cons of each staining method and depending on the object of study, it may be most interesting to combine several techniques, as previously mentioned.

On the other hand, immunohistochemistry provides more specific information on the molecular changes in OA, showing a decrease in structural components such as type II collagen, an increase in degradative enzymes (MMP-13, MMP-3), apoptosis (caspase-3), and markers of hypertrophy (COL-X). Therefore, it highlights the need to identify new biomarkers that allow earlier detection and a better understanding of the pathological mechanisms of OA.

## Figures and Tables

**Table 1:** Results of the rabbit studies are included.

Author/Year	Immunohistochemistry	Histology
Pelletier et al., 2003 [20]	MMP-1	Safranin O
Kobayashi et al., 2005 [21]	MMP-1	Safranin O–Fast Green
Tibesku et al., 2005 [22]	CD44v6	Safranin O
Jean et al., 2008 [23]	GLAST, GLT-1	HE, Safranin O–Fast Green
Shirai et al., 2011 [24]	MMP-13, IL-1β, COL-X, VEGF, RANKL	HE, Safranin O
Zhang et al., 2011 [25]	MMP-13, BMP-2	HE, Safranin O
Jansen et al., 2012 [26]	VEGF	HE, Safranin O
Zhou et al., 2015 [27]	COL-I, COL-II	Safranin O–Fast Green, Sirius Red
Zhou et al., 2016 [28]	β-Catenin, Shh, Gli-2	HE, Safranin O–Fast Green
Akhtar et al., 2017 [6]	Caspase-3, p85	Safranin O–Fast Green
Luo et al., 2020 [29]	VEGF	HE
Shi et al., 2020 [30]	COL-II	Safranin O–Fast Green
Fang et al., 2021 [31]	COL-II	HE, Toluidine Blue
Hossain et al., 2021 [32]	MMP-1	HE, Safranin O, Masson’s trichrome
Liu et al., 2021 [33]	Bax, BcL, Caspase-3	HE, Safranin O–Fast Green, Toluidine Blue
Meng et al., 2021 [34]	COL-I, COL-II, MMP-3	HE, Safranin O–Fast Green, Toluidine Blue
Hossain et al., 2022 [35]	MMP-1	HE, Safranin O, Masson’s trichrome
Chen et al., 2022 [36]	Caspase-3, Caspase-9, COL-II	HE
Wang et al., 2022 [37]	BMP-2, SOX-9	HE

**Table 2:** Results of the rat studies are included.

Author/Year	Immunohistochemistry	Histology
Aktas et al., 2011 [38]	MMP-3	HE
Moon et al., 2012 [39]	MMP-13, iNOS, Nitrotyrosine	HE, Safranin O–Fast Green, Toluidine Blue
Lee et al., 2013 [40]	MMP-13, iNOS, RAGE	HE, Safranin O–Fast Green, Toluidine Blue
Wen et al., 2013 [41]	p38, JNK, phospho-ERK	HE
Di Rosa et al., 2014 [42]	CHI3L1, CHIT1	HE
Iijima et al., 2015 [43]	COL-II	Toluidine Blue
Iijima et al., 2016 [44]	COL-II	Toluidine Blue
Assis et al., 2016 [45]	IL-1β, Caspase-3, MMP-13	HE
Wen et al., 2016 [46]	TGF-β1	HE, Safranin O–Fast Green
Murata et al., 2017 [47]	TNF-α, IL-1β, Caspase-3	Safranin O–Fast Green, Toluidine Blue
Wei et al., 2017 [48]	LC3B	HE, Toluidine Blue
Isaka et al., 2017 [49]	COL-II, MMP-13	Toluidine Blue
Chen et al., 2018 [50]	COL-II, COL-X	Safranin O–Fast Green
Yan et al., 2018 [51]	MMP-13, COL-II, COL-X	HE, Safranin O–Fast Green, Alcian Blue/Hematoxylin
Jacer et al., 2018 [52]	COL-II	HE, Toluidine Blue
Bei et al., 2019 [53]	COL-II, MMP-3	HE, Safranin O–Fast Green
Yan et al., 2019 [54]	MMP-13, COL-II	HE, Safranin O–Fast Green, Alcian Blue/Hematoxylin
Ping et al., 2020 [55]	MMP-13, ADAMTS-5, COL-X, COL-II, TGF-β1, Aggrecan	Toluidine Blue
Wang et al., 2020 [56]	MMP-13, COL-II	HE, Safranin O–Fast Green
Yang et al., 2020 [57]	COL-II, MMP-13, RUNX2	HE, Toluidine Blue
Song et al., 2021 [58]	MMP-3, MMP-13, ADAMTS-4, Aggrecan, COL-II	Safranin O–Fast Green
Luo et al., 2021 [59]	COL-I, COL-II, MMP-8, MMP-9	Safranin O–Fast Green
Zhu et al., 2021 [60]	COL-II, COL-X	HE, Toluidine Blue
Chen et al., 2021 [61]	COL-II, COL-X, MMP-13	HE, Safranin O–Fast Green
Yan et al., 2022 [62]	MMP-13, COL-II, COL-X	HE, Safranin O–Fast Green, Alcian Blue/Hematoxylin
Barreto et al., 2022 [63]	COL-I,I IL-1β	Safranin O–Fast Green
Gökdemir et al., 2022 [64]	BPM-2, NF-κb	HE, Safranin O–Fast Green, Toluidine Blue
Song et al., 2022 [65]	MMP-3, MMP-13	Safranin O–Fast Green
Chen et al., 2022 [36]	COL-II, MMP-13, ADAMTS-5	HE, Safranin O–Fast Green
Chen et al., 2023 [66]	COL-II, MMP-13, Aggrecan, ADAMTS-4	HE, Safranin O–Fast Green
Sun et al., 2023 [67]	Ki67	HE, Safranin O–Fast Green
Han et al., 2024 [68]	COL-II, MMP-13	HE, Toluidine Blue
Haleem et al., 2024 [69]	COL-II, PCNA	HE, Alcian Blue, Masson’s trichrome
Lee et al., 2024 [70]	COL-II, MMP-9, Aggrecan, IL-1β	HE, Safranin O–Fast Green
Guo et al., 2024 [71]	COL-II, IL-6, NF-κb, p65	HE, Safranin O–Fast Green, Toluidine Blue
Giunta et al., 2024 [72]	PACAP	HE, Toluidine Blue

## Data Availability

Not applicable.

## Abbreviations

The following abbreviations are used in this manuscript:

AC	Articular Cartilage
ECM	Extracellular Matrix
COL-II	Type II Collagen
SZ	Superficial Zone
MZ	Middle Zone
DZ	Deep Zone
CC	Calcified Cartilage
SB	Subchondral Bone
OA	Osteoarthritis
MMPs	Matrix Metalloproteinases
IL-1β	Interleukin 1β
TNF-α	Tumor Necrosis Factor α
WOS	Web Of Science
HE	Hematoxylin–Eosin
SO	Safranin O
SO-FG	Safranin O–Fast Green
TB	Toluidine Blue
MT	Masson’s Trichrome
COL-X	Type X Collagen
iNOS	Inducible Nitric Oxide Synthase
RAGE	Receptor for Advanced Glycation End Products
NF-κB	Nuclear Factor Kappa
BMP-2	Bone Morphogenetic Protein 2
PACAP	Pituitary Adenylate Cyclase-Activating Polypeptide
COL-I	Type I Collagen
MMP-8	Matrix Metalloproteinase 8
MMP-9	Matrix Metalloproteinase 9
ADAMTS-4/5	A Disintegrin And Metalloproteinase with Thrombospondin Motifs 4/5
TGF-β1	Transforming Growth Factor Beta 1
PCNA	Proliferating Cell Nuclear Antigen
LC3	Microtubule-Associated Protein 1A/1B-Light Chain 3
MIA	Sodium Monoiodoacetate
